# Deep Reinforcement Learning for Real-World Humanoid Robot Locomotion Control with Automatic Reward Learning

**DOI:** 10.34133/research.1123

**Published:** 2026-02-06

**Authors:** Renzhi Lu, Jie Wang, Zonghe Shao, Ruijuan Chen, Lijun Zhu, Yuzhi Jiang, Yunyi Pang, Dongfang Liang, Yang Shi, Han Ding

**Affiliations:** ^1^ School of Artificial Intelligence and Automation, Key Laboratory of Image Processing and Intelligent Control, Engineering Research Center of Autonomous Intelligent Unmanned Systems, Chinese Ministry of Education, Huazhong University of Science and Technology, Wuhan 430074, China.; ^2^ School of Artificial Intelligence and Automation, Huazhong University of Science and Technology, Wuhan 430074, China.; ^3^ Research Center for Applied Mathematics and Interdisciplinary Science, School of Mathematics and Statistics, Wuhan Textile University, Wuhan 430200, Hubei, China.; ^4^ State Key Laboratory of Digital Manufacturing Equipment and Technology, Huazhong University of Science and Technology, Wuhan 430074, China.; ^5^ Department of Engineering, University of Cambridge, Cambridge CB2 1PZ, UK.; ^6^ Department of Mechanical Engineering, University of Victoria, Victoria, BC, V8W 3P6, Canada.

## Abstract

Humanoid robots possess the potential to solve complex problems across diverse environments, such as nuclear-contaminated zones, epidemic-affected areas, and extraterrestrial missions. However, a humanoid robot is an inherently complex system that integrates multiple disciplines, including perception, mechanical design, materials science, and motion control, each of which requires comprehensive and in-depth investigation. Among these aspects, motion control plays a crucial role, as it directly determines the robot’s motion accuracy, stability, and flexibility. In recent years, with the rapid evolution of graphics-processing-unit-based parallel computing and high-fidelity simulation environments, various deep-reinforcement-learning (DRL)-based approaches have been proposed to achieve precise and robust motion control due to its flexibility and adaptability in uncertain and dynamic environments. However, the inherent complexity and uncertainty of real-world tasks pose substantial challenges when designing effective reward functions for DRL agents. Most current methods typically rely on manually engineered or externally tuned reward signals and therefore require considerable domain expertise, associated with considerable human efforts and a long convergence time; these issues may even trigger mission failure. This work proposes an automatic reward learning method to derive reward functions for DRL in humanoid robot locomotion control. Specifically, a bilevel optimization framework is developed to enable automatic reward learning during policy learning. The reward learning mechanism in the upper level adaptively constructs and optimizes the reward function. The DRL framework in the lower level learns the locomotion control policy using the learned reward function. Three sets of experiments are conducted to verify the effectiveness of the proposed approach: training soft actor-critic and proximal policy optimization agents in MuJoCo environments, training the proximal policy optimization agent in a humanoid robot environment built with Isaac Lab, and transferring the agent to the real-world Unitree G1 humanoid robot through sim-to-real. The experimental results demonstrate that the proposed automatic reward learning method substantially improves learning efficiency and achieves superior performance to manually designed reward functions in both simulation and real-world deployment. By enhancing the success rate of transferring control policies from simulation to real-world humanoid robots, this approach provides a promising pathway toward accelerating the deployment of stable and adaptive humanoid robots in practical applications.

## Introduction

Benefiting from their form and joint architecture modeled on human anatomy, humanoid robots are endowed with the potential for humanlike flexibility [1–5], enabling them to perform complex locomotion and tasks in diverse environments, including traversing rugged terrains [6–9] and carrying heavy loads [10,11]. Even in hazardous environments such as nuclear contamination zones, epidemic areas, and extraterrestrial exploration missions, humanoid robots can be deployed to perform complex tasks, effectively preventing humans from being exposed to harmful substances [12–16]. However, as a highly integrated and complex system, a humanoid robot relies on multiple subsystems, including perception enabled by sensors, well-optimized mechanical design, material selection adapted to specific working environments, and accurate as well as efficient motion control algorithms [17–20]. Each of these aspects is essential for leveraging robotic capabilities across various fields. Among them, motion control stands at the core of humanoid capability, as it directly governs the robot’s ability to achieve dynamic stability, energy-efficient movement, and natural interaction with the environment [21–23]. Understanding and improving motion control not only determine a humanoid’s locomotion quality but also serve as a foundation for the integration of perception, planning, and learning in embodied intelligence systems.

To achieve high-performance locomotion control in humanoid robots, a variety of control paradigms have been explored, including model-based and learning-based methods. Model-based methods [9,24–28] provide reliable low-level joint regulation and whole-body motion control by leveraging explicit representations of robot dynamics. However, these approaches typically require extensive model tuning and remain sensitive to real-world uncertainties and computational constraints. These challenges have spurred growing interest in learning-based approaches [29], particularly deep reinforcement learning (DRL) [30], which offers a paradigm shift from handcrafted control to autonomous policy optimization.

In recent years, DRL has emerged as a promising framework for humanoid locomotion and whole-body control [31–34]. By learning control policies from scratch through exploratory strategies, DRL enables robots to acquire diverse motion skills efficiently in simulated environments and potentially transfer robust control policies to the real world. For example, Li et al. [32] introduced a dual-history architecture that leverages both the long-term and short-term input–output histories of the robot, achieving strong performance across multiple locomotion skills. Rodriguez and Behnke [35] introduced a curriculum-learning-based DRL framework that enables a single policy to acquire omnidirectional locomotion skills for humanoid robots. Haarnoja et al. [36] employed distributed maximum a posteriori policy optimization to train a policy in simulation, which was subsequently transferred to the real miniature humanoid robot OP3, enabling it to achieve robust and dynamic performance in a simplified one-versus-one soccer game and to exhibit the capability of anticipating ball movements. Baltes et al. [37] introduced a hierarchical reinforcement learning (RL) algorithm based on the deep deterministic policy gradient, enabling a dual-arm humanoid robot to autonomously type on arbitrary keyboards. These examples clearly demonstrate that DRL can effectively enable humanoid robots to acquire diverse skills such as playing a soccer game and typing on arbitrary keyboards, thereby further illustrating its capacity to learn different skills across different environments.

However, the performance and stability of DRL-based control policies largely hinge on the design of the reward function, which serves as the fundamental learning signal guiding policy optimization [38–42]. A well-designed reward function can accelerate policy convergence, improve motion stability, and enhance overall control efficiency. Designing such a reward function for humanoid locomotion, however, is particularly challenging. Humanoid robots possess high-dimensional, dynamically coupled structures that require the coordinated regulation of upper–lower body movements, hierarchical motor skills, and angular momentum [43]. These complexities make the reward landscape highly nonconvex and the exploration process unstable, often resulting in inefficient learning or divergent behaviors [31,44,45]. Despite extensive research, most existing reward functions remain handcrafted, relying heavily on expert knowledge and manual tuning. Such manually engineered rewards are often task specific, prone to suboptimal weighting between objectives, and highly sensitive to hyperparameter choices, issues that severely limit the scalability and generalization of DRL algorithms in complex control settings [46–48]. To address the challenge of specifying meaningful rewards, Zhang et al. [49] employed a hierarchical double deep Q-network-based hierarchical path planning method that still relies on carefully designed composite reward shaping to achieve stable convergence. Similarly, in quadruped locomotion, FootStep Reward was introduced to enable gait generation and transitions, but this approach also required extensive reward engineering [50].

Although these studies mark progress in task-specific reward formulation, researchers argue that automatically designing reward functions can substantially facilitate the application of DRL to humanoid robot control and improve the overall performance of the control process [51]. Automating reward function design in DRL has been pursued through 2 major directions. One relies on external information, either by reconstructing rewards from expert demonstrations via inverse RL [52–54] or by interactively refining rewards through human-in-the-loop active reward learning [53,55,56]. Both approaches have shown notable gains in robotic control tasks [56,57], yet each suffers from practical limitations: inverse RL demands high-fidelity expert trajectories [58], and active reward learning depends on frequent human intervention—conditions that are rarely feasible in real-world robot applications [59]. As a result, heavy reliance on external supervision remains a key barrier to the broader deployment of DRL in robotics [60]. An alternative line of research moves away from external guidance and instead derives reward signals directly from the agent’s own behavioral patterns [61]. Within this category, intrinsic-motivation-based methods constitute the dominant class [62], typically constructing rewards from heuristic indicators or task-agnostic measures. Despite extensive progress in automatic reward design, a general and adaptive framework for automatic reward construction remains a promising open problem [39,44,63,64].

Therefore, to address the challenge of reward function design in humanoid locomotion control, this work proposes a novel automatic reward learning framework grounded in a bilevel optimization formulation. This approach casts reward learning as a meta-learning problem, enabling the agent to jointly optimize both the control policy and its underlying reward signal through iterative interactions between 2 levels. At the lower level, a DRL agent optimizes its locomotion policy within a partially observable Markov decision process (POMDP), using reward signals provided by the upper level. The policy is trained using policy gradients based on partial observations, while the reward function evaluates policy behavior using full-state information, implicitly guiding the agent toward globally coherent motor strategies. At the upper level, the reward function is learned via the proposed meta-gradient algorithm that seeks to maximize the DRL agent’s long-term performance. The 2 levels operate in an alternating optimization scheme, wherein the reward function and policy are refined in tandem. This results in a feedback loop: as the policy evolves, the reward adapts to reinforce informative behavioral features, effectively shaping the learning landscape. Through this bilevel framework, the need for manual reward engineering is reduced, thereby enabling the autonomous learning of reward structures that align with both task objectives and agent capabilities. To validate the proposed framework, extensive experiments are conducted in both the MuJoCo and Isaac Lab humanoid locomotion environments, comparing against manually designed reward functions. Results show that the automatically learned rewards consistently accelerate policy convergence and yield superior control performance. Furthermore, the policy trained in Isaac Lab is successfully transferred to the real-world Unitree G1 humanoid robot via sim-to-real deployment, demonstrating robust locomotion without further fine-tuning. In brief, this work establishes a scalable and generalizable reward optimization paradigm for humanoid control, laying the foundation for more autonomous, adaptive, and interpretable DRL in real-world robotics. By reducing reliance on manual heuristics and enhancing the success rate of transferring control policies from simulation to the real world, the proposed approach paves the way for the next generation of embodied intelligence systems.

### Framework overview

**Fig. 1.**
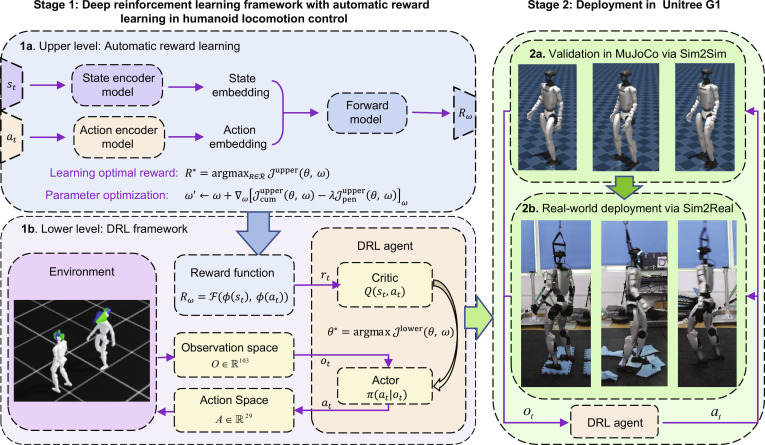
Overview of the proposed automatic reward learning framework for deep reinforcement learning (DRL) in humanoid locomotion control. In stage 1, an automatic reward learning mechanism is designed based on a bilevel optimization framework. At the upper level, an adaptive reward function is discovered and optimized to guide the learning process in the lower-level DRL module. A bilevel alternating optimization approach is employed to jointly refine the control policy and the reward function, thereby enhancing policy efficiency and stability during simulation-based training. In stage 2, the trained DRL agent is deployed on a real-world humanoid robot (Unitree G1) through sim-to-sim validation and sim-to-real transfer.

An overview of the proposed framework is illustrated in Fig. 1. For the humanoid locomotion control task, we employ an automatic reward learning method embedded within a DRL framework (stage 1), followed by real-world deployment of the learned agent on the humanoid robot Unitree G1 for experimental validation (stage 2). In stage 1, we formulate a bilevel optimization framework to enable automatic reward learning during policy learning. At the upper level, a reward learning mechanism adaptively constructs and optimizes the reward function. At the lower level, a DRL framework learns the locomotion control policy in a simulated environment using the learned reward function. An alternating optimization scheme coordinates the 2 levels: the upper level evaluates and refines the reward function based on the agent’s performance, while the lower-level agent updates its control policy using the newly generated rewards. This iterative interaction continues until both the reward function and the policy converge. In stage 2, the trained DRL agent is transferred to the real-world Unitree G1 robot through sim-to-sim validation and sim-to-real transfer. We first transfer the learned locomotion control policy from the Isaac Lab simulator to the MuJoCo simulation environments using a sim-to-sim approach for validation. Subsequently, the policy is deployed to the real-world Unitree G1 humanoid robot via a sim-to-real method, and its performance is evaluated across various terrains.

## Results

In this section, we analyze the performance of the automatic reward learning approach in both simulated and real-world humanoid robot locomotion control tasks. Our framework, validated across simulated and real-world humanoid platforms, shows an unprecedented ability to autonomously learn reward structures that generalize across environments and embodiments. The resulting control policies exhibit remarkable stability, efficiency, and adaptability, contributing to the advancement of learning-driven motion control.

### Validation in continuous-control simulated robots

#### Challenging articulated robot locomotion for evaluating generalizable reward learning

Mastering locomotion for articulated robots in continuous-control settings remains a central challenge, due to the high degrees of freedom, intricate joint coordination, and complex contact dynamics required for stable movement. These environments demand DRL algorithms that are not only robust and sample efficient but also capable of learning reward structures that generalize across robots with varying morphologies and motion objectives. Evaluating in such benchmarks provides a rigorous test of a method’s scalability and effectiveness in realistic robot learning scenarios.

#### Comprehensive benchmarks on articulated robot locomotion

To thoroughly assess our approach, we evaluate the proposed automatic reward learning method on 4 representative MuJoCo environments [65]: Reacher-v2, Hopper-v2, HalfCheetah-v2, and Humanoid-v2. These environments span a broad spectrum of articulated robot morphologies and control difficulties, each requiring precise multijoint coordination for successful locomotion or manipulation. All experiments are conducted using standardized policy architectures and hyperparameters, ensuring fair and direct comparison between methods.

#### Consistent improvements with PPO and SAC

**Fig. 2.**
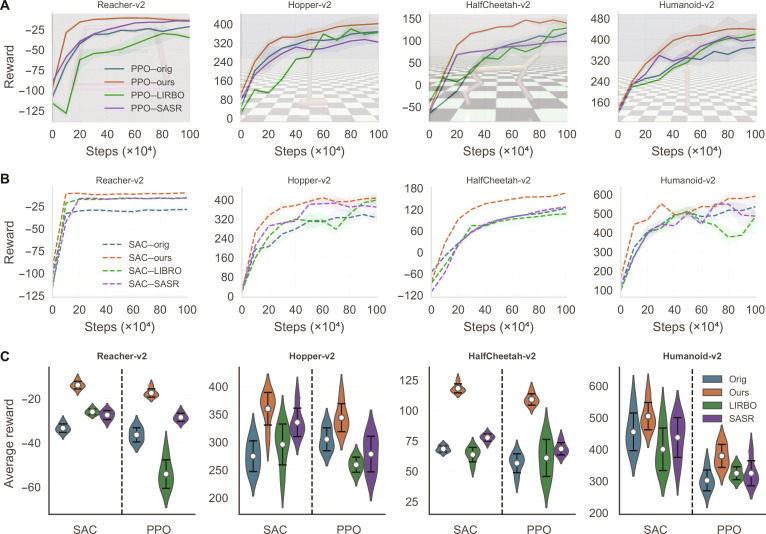
Performance comparison of DRL algorithms across 4 MuJoCo environments. (A) Learning curves for proximal policy optimization (PPO) agents with different reward functions on Reacher-v2, Hopper-v2, HalfCheetah-v2, and Humanoid-v2 tasks. For each environment and algorithm, the average reward (*y*-axis) is plotted as a function of training steps (×10^4^). The solid lines indicate the mean performance over 5 independent runs, while the shaded regions represent ±1 standard deviation. (B) Learning curves for soft actor-critic (SAC) agents across various tasks using different reward functions. (C) Average testing rewards and distribution (across seeds) for PPO and SAC agents after training. Each violin plot shows the distribution of final rewards over 5 runs, with embedded white dots and error bars indicating the mean ± standard deviation. Individual experiment results are overlaid as semitransparent points. Orange indicates our method, blue indicates the original reward, green indicates LIRBO, and purple indicates SASR.

To demonstrate the robustness and generality of our automatic reward learning approach, we compare it with baseline rewards using both proximal policy optimization (PPO) and soft actor-critic (SAC) algorithms. The baseline rewards include the original reward functions from OpenAI Gym and 2 reward learning methods, LIRBO [66] and SASR [67]. LIRBO reformulates intrinsic reward learning in RL as a bilevel optimization problem and leverages self-tuning networks with conditional layer normalization to adaptively learn intrinsic rewards. SASR realizes a self-adaptive success-rate-based reward shaping framework that uses kernel density estimation with random Fourier features to model a Beta distribution over success rates, enabling an automatic exploration–exploitation trade-off and improving sample efficiency and stability in sparse-reward RL tasks. As shown in Fig. 2A and B, our method (orange) consistently accelerates learning and achieves higher episodic rewards than the other baseline methods across all 4 tasks. For both PPO and SAC agents, the reward curves exhibit faster convergence and higher final performance, particularly in the more challenging locomotion tasks such as HalfCheetah-v2 and Humanoid-v2. SASR and LIRBO exhibit similar overall performance, but SASR attains better results when paired with the PPO agent. Each curve reports the mean and standard deviation across 5 independent runs.

#### Superior policy generalization and stability

To further assess the generalization ability of learned policies, we evaluate trained agents over multiple seeds and environments. Figure 2C summarizes that our approach yields higher average test-time rewards and reduced variance for both SAC- and PPO-trained policies. This advantage is most evident in high-dimensional tasks like Humanoid-v2, where our method substantially outperforms the baseline in terms of both the mean and consistency of policy performance.

#### Enhanced sample efficiency across diverse morphologies

Together, these results highlight the versatility and effectiveness of our approach in learning reward functions that enable faster, more stable, and more robust policy learning for articulated robots. The consistent gains observed across environments and algorithms underscore the potential of automatic reward learning in advancing DRL for complex robotic systems.

### Scaling to full-body humanoid control in simulation

#### From articulated control to full-body humanoid locomotion

Extending from articulated MuJoCo robots to a full-body humanoid introduces substantially greater complexity in dynamics, control coordination, and reward design. Unlike lower-dimensional agents such as Hopper or HalfCheetah, a humanoid robot must maintain whole-body balance, manage coupled joint dynamics, and coordinate upper- and lower-limb motions under high-dimensional control spaces. These challenges amplify the difficulty of both policy optimization and reward shaping, making humanoid control an ideal test bed for assessing the generality and robustness of our automatic reward learning framework.

#### Experimental setup in Isaac lab

We evaluate the proposed method in NVIDIA Isaac Lab using a simulated 29-degree-of-freedom (29-DOF) Unitree G1 humanoid robot with 4,096 parallel environments. The simulation environment, developed by Unitree Robotics, provides standardized physical modeling and a baseline reward function for locomotion. Our experiments adopt the same PPO-based policy architecture and hyperparameter settings as in MuJoCo to ensure fair comparison and direct scalability assessment. Training is conducted on flat terrain to focus on gait learning performance while maintaining compatibility with subsequent real-world deployment. Isaac Lab, built upon PhysX and Isaac Sim, offers graphics-processing-unit-accelerated parallel simulation and native DRL integration, enabling large-scale training of complex humanoid control tasks with high physical fidelity.

#### Superior performance of reward components during policy learning

**Fig. 3.**
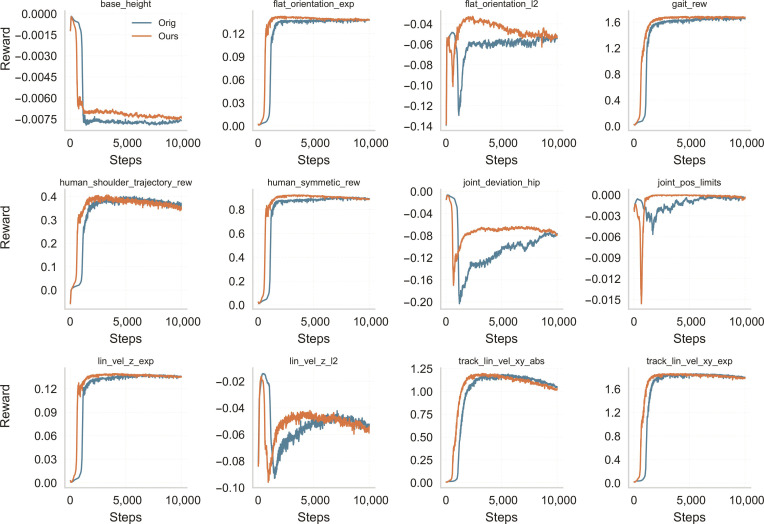
Performance comparison between manually designed reward functions and automatic reward learning method across 12 reward components governing humanoid locomotion in Isaac Lab. The components span velocity-tracking accuracy, whole-body balance, motion smoothness, safety constraints, and gait stability, providing a fine-grained characterization of how each reward formulation shapes policy behavior throughout learning.

As shown in Fig. 3, the automatic reward learning method yield more stable improvements in base height and orientation regulation, stronger enforcement of joint safety limits, and smoother and more symmetric gait behaviors, across the 12 reward components. Our method also achieves higher fidelity in vertical velocity tracking. Overall, the comparison shows that the automatic reward learning method provides more balanced and coherent guidance across stability, safety, and locomotion objectives than manually engineered rewards.

#### Stabilized gait performance in humanoid locomotion

**Fig. 4.**
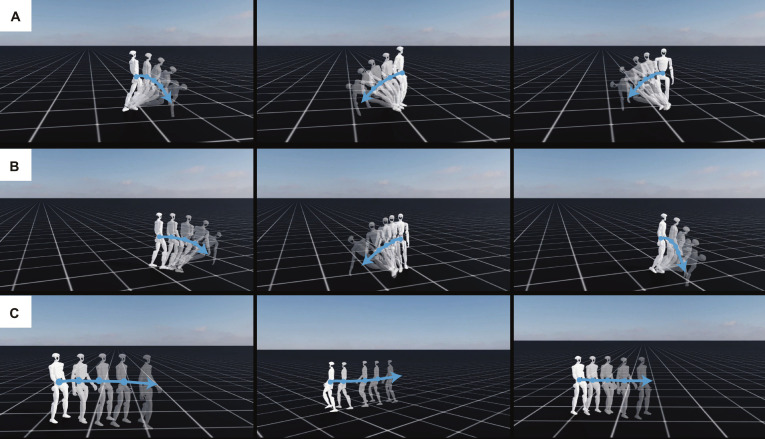
Snapshots of the humanoid robot’s gait, which is controlled by PPO trained with manually designed reward functions at different training stages. The robot struggles with stability and coordination in early training stages, frequently exhibiting leaning, unbalanced posture, and falling. Only after extensive training does the policy begin to demonstrate relatively upright walking behavior. Results illustrate the slow convergence and instability associated with handcrafted reward designs in high-degree-of-freedom (high-DOF) humanoid locomotion tasks. (A) 500 iterations. (B) 1,000 iterations. (C) The final converged policy.

**Fig. 5.**
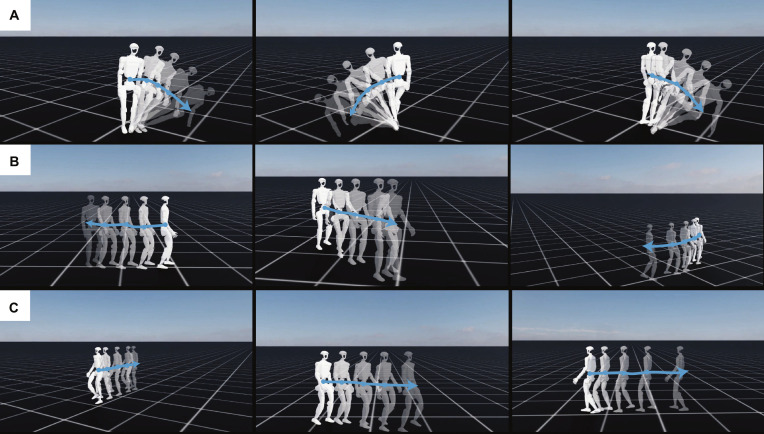
Snapshots of the humanoid robot’s gait, which is controlled by PPO trained with the proposed reward learning approach. (A) 500 iterations. (B) 1,000 iterations. (C) The final converged policy.

To qualitatively evaluate the evolution of locomotion behaviors, we analyze humanoid gait patterns learned in simulation using visual observations at different training stages. Figures 4 and 5 present representative frames from PPO agents trained with the handcrafted reward functions and the proposed automatic reward learning method, respectively, captured at 500 iterations, 1,000 iterations, and the final converged policy. As shown in Figs. 4A and 5A, at 500 iterations, both policies show limited locomotion ability and the agents exhibit unstable or ineffective movement patterns. However, by 1,000 iterations, a clear divergence emerges in Figs. 4B and 5B that the baseline-trained PPO continues to struggle with initiating stable forward motion and maintaining balance, whereas our method yields a humanoid capable of executing forward stepping with coordinated posture and reduced instability. In the final training stage, our approach consistently produces smooth, symmetric gait cycles with upright torso alignment, effective step alternation, and reduced lateral oscillation, as shown in Fig. 5C. These visual results confirm that our framework accelerates the emergence of meaningful locomotion skills, substantially outperforming manually designed rewards in both learning speed and gait stability. The policy trained via automatic reward learning method not only converges faster but also exhibits more humanlike and robust walking behaviors in the Isaac Lab simulator. Such improvements lay a strong foundation for successful sim-to-real transfer to the real Unitree G1 robot, where early gait stability is crucial for safe and efficient real-world deployment.

#### Effect of the alignment coefficient

We study the effect of the regularization coefficient *λ* by varying its initial value in the Isaac Lab environment while keeping the annealing schedule fixed. This coefficient controls the alignment strength between the learned and prior rewards. As shown in Table 1, different initial values of *λ* have little effect on the final performance, mainly influencing early-stage convergence: larger values accelerate and stabilize early training, while smaller values allow more exploration. Overall, the final rewards are robust to *λ*, indicating that performance gains are not sensitive to this hyperparameter.

**Table 1.** Analysis of the alignment coefficient

Method	Reward in 1,000 steps	Best reward
Our method with *λ* = 20	103.6	123.6
Our method with *λ* = 10	68.8	122.3
Our method with *λ* = 5	47.1	124.0

### Real-world deployment and terrain generalization

To assess the real-world effectiveness and generalizability of our proposed automatic reward learning framework, we deploy the learned policy onto the real-world Unitree G1 humanoid robot through a sim-to-real method. The policy is trained entirely in simulation using Isaac Lab, without any real-world fine-tuning or human demonstrations.

#### Unitree G1 humanoid robot

Unitree G1 is a general-purpose humanoid robot developed by Unitree Robotics, standing approximately 1.32 m tall and weighing about 35 kg. Its structure is divided into an upper and a lower body, providing a total of 29 DOFs. Each arm offers 7 DOFs, including the shoulder, upper arm, elbow, and wrist joints, while each leg provides 6 DOFs, encompassing the hip (pitch, roll, and yaw), knee, and ankle joints. Unitree G1 has been widely employed as a humanoid platform for research in locomotion control [68,69].

#### Sim-to-sim transfer as an intermediate validation

**Fig. 6.**
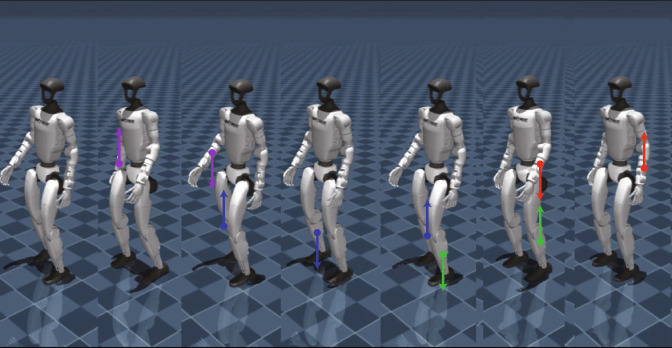
Gait results from sim-to-sim experiments in the MuJoCo environment. Red, purple, green, and blue arrows indicate the motion trajectories of the left arm, right arm, left leg, and right leg, respectively. The locomotion trajectory shows a contralateral coordination pattern, with left-leg elevation accompanied by right-arm lift, consistent with human locomotion.

Before directly transferring the policy to real-world robot, we first perform a sim-to-sim validation to examine the cross-platform transferability and structural stability of the learned policy. Specifically, we migrate the policy from the Isaac Lab simulator to the MuJoCo simulator, which has different contact dynamics and physics engines. This intermediate validation enables us to isolate policy behavior from hardware noise and assess gait robustness in a distinct simulated environment. As shown in Fig. 6, the policy, trained entirely with automatic reward learning, exhibits smooth walking gaits, preserved posture control, and consistent step alternation in MuJoCo, suggesting strong cross-simulator generalization. This sim-to-sim result further confirms that the learned reward function encapsulates transferable locomotion priors, independent of simulation-engine-specific dynamics.

#### Sim-to-real transfer

We used the Unitree G1 simulation environment developed by Unitree Robotics, based on our custom G1 robot configuration and reward function design, and successfully migrated it to the Isaac Lab platform, enabling support for 29-DOF control. This procedure guarantees consistency between the state information used during policy training and the sensory inputs available on the physical platform, thereby promoting the learning of more stable control policies. The resulting policies can subsequently be deployed on the real-world Unitree G1 humanoid robot to achieve robust and reliable operation.

#### Robust real-world locomotion on diverse terrains

**Fig. 7.**
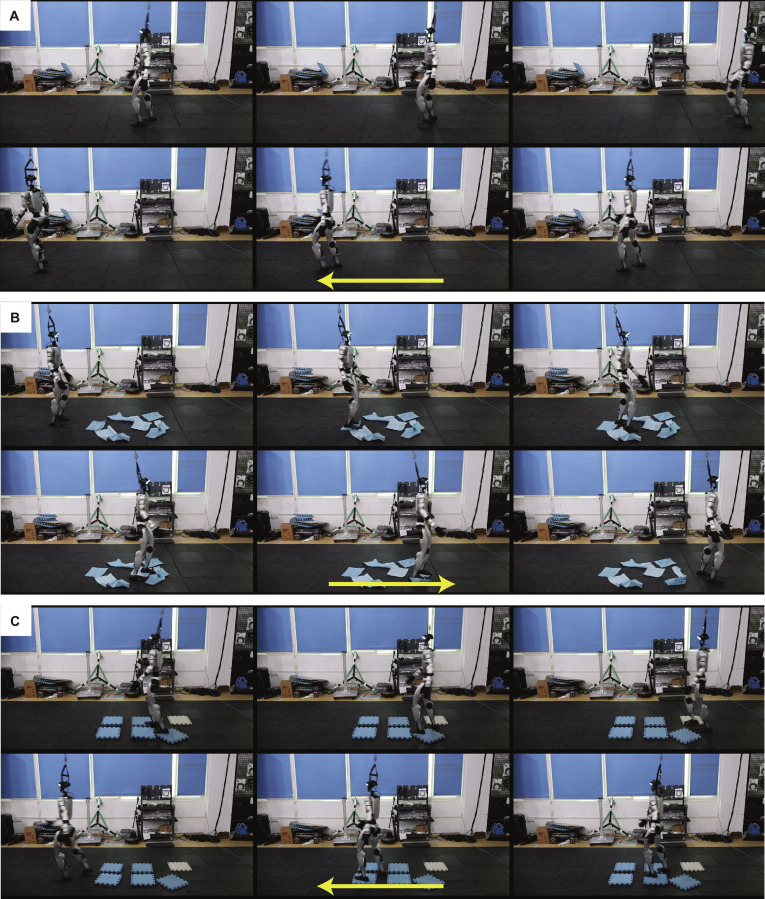
Snapshot of real motion. We transfer the trained policies from simulation to 3 real-world scenarios corresponding to the 3 terrain types. (A) Walking on a flat rubber terrain from right to left. (B) Walking on a terrain covered with scattered garbage from left to right. (C) Walking on a terrain covered with crawl pads from right to left.

We evaluate the learned control policy in 3 real-world environments, namely, a flat rubber surface (Fig. 7A), a terrain covered with randomly scattered garbage (Fig. 7B), and a terrain cluttered with crawl pads (Fig. 7C). These terrains present increasing levels of complexity and surface irregularity, offering a rigorous test for control robustness and adaptability. Upon deployment, the policy demonstrates stable and symmetric gait patterns across all 3 real-world terrains. On flat ground, the robot exhibits smooth forward walking with minimal torso sway and consistent footfall timing. When traversing terrain covered with scattered garbage or crawl pads, the robot maintains balance, forward progression, and stepping regularity, despite encountering surface compliance, contact variation, and mild disturbances.

#### Quantitative evaluations

To evaluate energy efficiency, we use 2 physically meaningful surrogate metrics: a torque-based energy metric and a mechanical effort metric combining squared torques, velocities, and accelerations. Compared to the baseline, our method achieves approximately a 12% reduction in energy consumption, as shown in Table 2. For the success rate, in both Isaac Lab and real-world experiments, success is defined as the humanoid robot traversing the terrain without falling; under this criterion, our method achieves 100% success in simulation and 90% in real-world trials, with the remaining failures caused by hardware limitations rather than the learned policy.

**Table 2.** Estimating relative energy consumption through torque

Metrics	Baseline	Ours	Reduction rate
Torque-based energy	0.1102	0.0967	12.2%
Mechanical effort	0.1284	0.1123	12.5%

Notably, all testing terrains are unseen during training, and the controller is deployed without any real-world fine-tuning, highlighting the zero-shot generalization ability of the learned policy. Throughout the trials, the robot remains upright and stable, showing no signs of falling, foot dragging, or joint saturation. These results confirm that the policies trained with automatically learned rewards can reliably transfer from simulation to the physical robot and maintain locomotion performance under moderate real-world variation.

## Discussion

This work introduces an automatic reward learning method for real-world humanoid robot locomotion control based on DRL. The proposed framework redefines how humanoid locomotion control reward functions can be learned rather than manually designed. By formulating reward learning as a bilevel meta-optimization problem, our approach enables a robot to autonomously infer what to optimize instead of being constrained by human-designed heuristics. This shift from fixed reward specification to adaptive objective learning represents a conceptual step toward self-optimizing robotic intelligence. Our experiments across both MuJoCo and Isaac Lab demonstrate that automatic reward learning consistently yields faster convergence, higher stability, and more natural gait emergence compared with manually defined rewards. More importantly, the framework generalizes across platforms, from simulated joint-level control to full-body humanoid motion, indicating that the reward learning method can adapt to different priors and encapsulate transferable control priors that remain valid across environments and embodiments. Beyond locomotion, the underlying principle of meta-learned reward structures offers a general mechanism for transferring learning objectives across tasks, morphologies, and embodiments. Because the proposed framework optimizes reward learning rather than fixed policies, the learned reward priors can serve as transferable behavioral abstractions, allowing knowledge from one type of robot or motion task to accelerate learning in another. For example, reward functions distilled from humanoid locomotion could provide meaningful inductive biases for legged or multilimbed robots and even inform manipulation or whole-body coordination tasks. This cross-domain generality highlights that automatic reward learning can unify DRL objectives across diverse robotic systems, moving toward a framework where robots share and evolve common notions of success. While the current study focuses on structured locomotion tasks, these findings suggest a pathway toward self-evolving robotic intelligence, in which robots continuously refine and exchange their reward representations to adapt to novel tasks and embodiments with minimal human intervention.

Future work will focus on improving scalability to complex multiagent and high-dimensional systems by developing decentralized reward optimization methods and incorporating structured or modular reward representations.

## Methods

### Problem formation

#### Partially observable Markov decision process

We formulate the humanoid control and decision-making process as a constrained POMDP $M_{\omega }=\left(S,O,A,P,R_{\omega },\gamma \right)$, where *S* denotes the full state space of the environment, O represents the partial observation space available to the agent, and *A* is the discrete action set. The transition probability $P\left(s^{\prime }|s,a\right)$ defines the underlying dynamics, and the discount factor $\gamma \in \left(0,1\right]$ regulates the contribution of future rewards. The reward function $R_{\omega }\left(s,a\right)$ is parameterized by ω and depends on the fully observable state–action pair, whereas the policy $\pi_{\theta }\left(a|o\right)$, parameterized by *θ*, relies only on partial observations. At each time step *t*, the environment provides a partial observation $o_{t}∼p\left(o_{t}|s_{t}\right)$, the agent samples an action $a_{t}∼\pi_{\theta }\left(a_{t}|o_{t}\right)$, and the environment transitions to the next state $s_{t+1}∼P\left(s_{t+1}|s_{t},a_{t}\right)$, yielding a reward signal $r_{t}=R_{\omega }\left(s_{t},a_{t}\right)$. This formulation allows the policy to operate under sensory uncertainty, while the reward model exploits full-state information to encode task-level structure and environmental context.

#### Action space

The action vector $a_{t}\in {\mathbb{R}}^{29}$ specifies the target positions of the G1 humanoid robot’s 29 actuated joints, spanning the lower body (hips, knees, and ankles), torso (waist yaw/roll/pitch), and upper body (shoulders, elbows, and wrists). These target configurations are generated by the actor network and tracked by a proportional-derivative controller, which converts the desired positions into joint torques for precise and stable motion execution.

#### Observation space

The policy observation vector $o_{t}\in {\mathbb{R}}^{103}$ consists of 3 components:

$$o_{t}=\left[c_{t},o_{t}^{\text{proprio}},a_{t-1}\right],$$ (1)

where $c_{t}\in {\mathbb{R}}^{7}$ is the gait command, $o_{t}^{\text{proprio}}\in {\mathbb{R}}^{67}$ contains proprioceptive signals (base angular velocity $\omega_{t}\in {\mathbb{R}}^{3}$, gravity $g_{t}\in {\mathbb{R}}^{3}$, commanded velocity $c\;v_{t}\in {\mathbb{R}}^{3}$, joint positions $\theta_{t}^{p}\in {\mathbb{R}}^{29}$, and joint velocities $\theta_{t}^{v}\in {\mathbb{R}}^{29}$), and $a_{t-1}\in {\mathbb{R}}^{29}$ is the previous action.

#### Full state

The full environmental state $s_{t}$, accessible to the reward and value functions, comprises all the policy observations plus additional privileged information that the policy cannot access directly. In our setup, this includes an egocentric elevation map $o_{t}^{\text{percept}}\in {\mathbb{R}}^{15\times 15}$, centered on the robot and sampled on a 0.1-m grid, as well as all components of $o_{t}$. Formally,

$$s_{t}=\left[c_{t},o_{t}^{\text{proprio}},o_{t}^{\text{percept}},a_{t-1}\right].$$ (2)

The structure of POMDP enables the reward model and the critic to leverage complete environmental information for evaluation and learning, while the policy remains constrained to partial observation, ensuring realistic decision-making under uncertainty.

#### Prior reward

Traditionally, prior knowledge of robot dynamics and control objectives is incorporated into the design of a handcrafted reward function, typically expressed as a weighted sum of multiple terms,

$$R^{†}\left(s,a\right)=\sum_{k}w_{k}r_{k}\left(s,a\right),$$ (3)

where each component $r_{k}$ encodes a specific control goal, such as tracking commanded base velocities, maintaining body stability, ensuring smooth and safe motion, and promoting humanlike gait characteristics. The corresponding weights $w_{k}$ are empirically tuned to balance tracking accuracy, safety, and motion naturalness.

The prior reward serves only as a weak and generic initialization that encodes basic physical and safety constraints, while the final reward is adaptively refined and learned through bilevel meta-optimization based on the agent’s own interaction and performance. In the following section, we introduce our automatic reward learning framework, which replaces handcrafted reward design with a data-driven mechanism that jointly optimizes the reward and policy through bilevel learning.

### Automatic reward learning

The autonomous reward learning process is formulated as a bilevel optimization problem in which the policy and the reward function coevolve through iterative interactions. At the lower level, the policy $\pi_{\theta }$ is optimized within the POMDP to maximize cumulative returns derived from the current reward model $R_{\omega }$. At the upper level, the reward function is updated to enhance the long-term utility of the learned policy while remaining grounded in the guidance provided by the prior $R^{†}$. In robotic control settings, it is often possible to construct such a prior reward based on domain knowledge or heuristic principles, reflecting intuitive notions of task success or stability. However, these manually designed priors are typically imperfect and may fail to capture the nuanced trade-offs required for optimal behavior. The goal of the autonomous learning process is therefore to learning a reward function $R_{\omega }$ that maximizes the long-term effectiveness of the policy—while constraining the learned reward to deviate minimally from the prior so as to preserve the reliability and interpretability inherent in the existing knowledge. This coupling naturally produces a feedback loop: as the policy improves its control behavior, the reward function adapts to emphasize the most informative aspects of the environment, further shaping the policy’s learning trajectory. Through this iterative process, the reward function becomes an emergent objective that reflects both environmental dynamics and desired behavioral semantics.

### Lower-level policy optimization

Given the current reward function $R_{\omega }$, the lower-level objective is to learn a policy that maximizes the expected discounted return,

$$J^{\text{lower}}\left(\theta ,\omega \right)=E_{o_{t},a_{t}∼\pi_{\theta },s_{t}∼M_{\omega }}\left[\sum_{t=0}^{\infty }\gamma^{t}R_{\omega }\left(s_{t},a_{t}\right)\right].$$ (4)

The policy gradient theorem provides the corresponding gradient of this objective:

$$\nabla_{\theta }J^{\text{lower}}\left(\theta ,\omega \right)=E_{\mu_{o,a}}\;\left[\nabla_{\theta }\text{log}\pi_{\theta }\left(a|o\right)\;Q_{R_{\omega }}^{\pi_{\theta }}\left(o,a\right)\right],$$ (5)

where $Q_{R_{\omega }}^{\pi_{\theta }}\left(o,a\right)$ represents the state–action value under partial observability and $\mu_{o,a}$ denotes $\left(o,a∼\pi_{\theta },s∼M_{\omega }\right)$. The policy is updated iteratively using gradient ascent [70],

θ′=θ+α⋅∇θJlowerθ,ω=θ+α⋅Eμo,a∇θlogπθaoQRωπθo,a, (6)

where *α* is the learning rate.

This lower-level optimization encourages the agent to refine its decision-making based on the partial observations available, while the reward function provides a full-state evaluation that implicitly guides the policy toward globally coherent behavior.

### Upper-level reward optimization

#### Upper-level reward objective

At the upper level, the central objective is to learn a reward function that enables the agent to learn effective and robust policies, while remaining consistent with available domain knowledge. In robotic control tasks, it is often possible to design a prior reward function $R^{†}$ based on physical intuition, heuristics, or task-specific criteria. However, such priors are inevitably limited and may overlook critical aspects of the behavior required for optimal performance [71,72]. Thus, our framework seeks to learn a new reward function $R_{\omega }$ that maximizes the expected long-term return of the policy [73] while drawing on reliable prior knowledge from $R^{†}$ to guide and regularize the learning process.

To formalize this, we define the upper-level objective as a combination of 2 components,

$$J^{\text{upper}}\left(\theta ,\omega \right)=J_{\mathrm{cum}}^{\text{upper}}\left(\theta ,\omega \right)-\lambda \;J_{\mathrm{pen}}^{\text{upper}}\left(\theta ,\omega \right),$$ (7)

where $J_{\mathrm{cum}}^{\text{upper}}$ encourages the learned reward to support policies that achieve high expected return under the prior reward and $J_{\mathrm{pen}}^{\text{upper}}$ penalizes deviations from the behavioral structure implied by the prior reward. Specifically, $J_{\mathrm{cum}}^{\text{upper}}$ is given by the expected cumulative return of the policy under the prior reward, while $J_{\mathrm{pen}}^{\text{upper}}$ quantifies the difference in advantage structure between the learned reward and the prior reward $R^{†}$. The coefficient λ>0 balances the pursuit of novel, effective rewards with the preservation of essential prior knowledge.

The first term, $J_{\mathrm{cum}}^{\text{upper}}$, is defined as the expected cumulative return of the policy under the prior reward function,

$$J_{\mathrm{cum}}^{\text{upper}}\left(\theta ,\omega \right)=E_{\pi_{\theta }∼M_{\omega }}\left[G_{R^{†}}^{\pi_{\theta }}\right],$$ (8)

where $G_{R^{†}}^{\pi_{\theta }}$ denotes the discounted sum of rewards collected by following policy $\pi_{\theta }$ in the environment governed by $R^{†}$. This component ensures that the learned reward remains anchored to established domain knowledge and does not incentivize degenerate behaviors.

The second term, $J_{\mathrm{pen}}^{\text{upper}}$, quantifies the degree to which the advantage function induced by the learned reward deviates from that of the prior reward,

$$J_{\mathrm{pen}}^{\text{upper}}\left(\theta ,\omega \right)=E_{\mu_{s,a}}\left[1-\frac{A_{R_{\omega }}^{\pi_{\theta }}\left(s,a\right)\cdot A_{R^{†}}^{\pi_{\theta }}\left(s,a\right)}{\left\|A_{R_{\omega }}^{\pi_{\theta }}\left(s,a\right)\right\|\left\|A_{R^{†}}^{\pi_{\theta }}\left(s,a\right)\right\|+\epsilon }\right],$$ (9)

where $A_{R^{†}}^{\pi_{\theta }}\left(s,a\right)$ is the advantage computed with respect to the prior reward $R^{†}$, $A_{R_{\omega }}^{\pi_{\theta }}\left(s,a\right)$ is the advantage under the learned reward, and ϵ is a small constant for numerical stability. This regularization constrains the optimization to favor reward functions whose advantage structures remain close to those prescribed by the prior, thus maintaining the reliability and interpretability of the learned reward.

#### Gradient formulation

To optimize the upper-level objective with respect to the reward parameters ω, we compute the gradient of $J^{\text{upper}}\left(\theta ,\omega \right)$ as follows:

$$\nabla_{\omega }J^{\text{upper}}\left(\theta ,\omega \right)=\nabla_{\omega }J_{\mathrm{cum}}^{\text{upper}}\left(\theta ,\omega \right)-\lambda \;\nabla_{\omega }J_{\mathrm{pen}}^{\text{upper}}\left(\theta ,\omega \right).$$ (10)

Next, we analyze the gradient of the cumulative return term $J_{\mathrm{cum}}^{\text{upper}}$ at the updated policy parameters $\theta^{\prime }$ by applying Eq. (6). The policy parameters $\theta^{\prime }$ implicitly depend on the reward parameters ω, as the policy is optimized under the guidance of the current reward. Therefore, the gradient of the upper-level objective with respect to ω is

$$\nabla_{\omega }J_{\mathrm{cum}}^{\text{upper}}\left(\theta =\theta^{\prime },\omega \right)=\frac{\partial }{\partial \omega }J_{\mathrm{cum}}^{\text{upper}}\left(\theta^{\prime },\omega \right)+\frac{\partial }{\partial \theta^{\prime }}J_{\mathrm{cum}}^{\text{upper}}\left(\theta^{\prime },\omega \right)\cdot \frac{d\theta^{\prime }}{\mathrm{d\omega}}.$$ (11)

The first term can be rewritten as

$$\frac{\partial }{\partial \omega }J_{\mathrm{cum}}^{\text{upper}}\left(\theta^{\prime },\omega \right)=E_{\mu_{s,a}^{\prime }}\left[Q_{R^{†}}^{\pi_{\theta^{\prime }}}\left(s,a\right)\cdot \frac{\partial \text{log}\pi_{\theta }\left(o,a\right)}{\partial \omega }\right],$$ (12)

where $\mu_{s,a}^{\prime }$ represents the state–action distribution under the policy $\pi_{\theta^{\prime }}$.

Based on the definition of $G_{R}^{\pi }$, the optimization for $\frac{\partial }{\partial \theta }J_{\mathrm{cum}}^{\text{upper}}\left(\theta^{\prime },\omega \right)$ can be defined as

$$\frac{\partial }{\partial \theta^{\prime }}J_{\mathrm{cum}}^{\text{upper}}\left(\theta^{\prime },\omega \right)=E_{\mu_{s,a}^{\prime }}\left[Q_{R^{†}}^{\pi_{\theta^{\prime }}}\left(s,a\right)\cdot \frac{\partial \text{log}\pi_{\theta^{\prime }}\left(o,a\right)}{\partial \theta^{\prime }}\right].$$ (13)

Considering that changes in the reward affect the policy parameters only indirectly through the policy update process, rather than directly modifying the parameters themselves, we can therefore assume that $\frac{\partial }{\partial \omega }J_{\mathrm{cum}}^{\text{upper}}\left(\theta^{\prime },\omega \right)=0$ and the term $\frac{d\theta^{\prime }}{\mathrm{d\omega}}$ can be expressed as follows:

$$\frac{d\theta^{\prime }}{\mathrm{d\omega}}=\alpha \cdot E_{\mu_{s,a}}\left[\frac{\partial Q_{R_{\omega }}^{\pi_{\theta }}\left(s,a\right)}{\partial \omega }\cdot \frac{\partial \text{log}\pi_{\theta }\left(o,a\right)}{\partial \theta }\right].$$ (14)

Under a finite action space, the policy is parameterized as a normalized action-preference function. By exploiting the relationship between the value function $V_{R_{\omega }}^{\pi_{\theta }}\left(s\right)$ and action-value functions and the linearity of the expectation operator, the summation and expectation collapse into a single expectation over state–action pairs, yielding the following:

∂∂θ′Jcumupperθ′,ω⋅dθ′dω=Eμs,a′QR†πθ′⋅∇θlogπθθ′⋅Eμs,a∇ωQRωπθω⋅∇θlogπθθ=Eμs,a′QR†πθ′s,a−VR†πθ′s⋅xs,a⋅Eμs,a∂∂ωQRωπθs,a−VRωπθs⋅xs,a, (15)

where $x_{s,a}$ is the feature vector of the state–action pair.

Therefore, we can obtain

$$\nabla_{\omega }J_{\mathrm{cum}}^{\text{upper}}\left(\theta ,\omega \right)=E_{\mu_{s,a}^{\prime }}\left[Q_{R^{†}}^{\pi_{\theta^{\prime }}}\left(s,a\right)-V_{R^{†}}^{\pi_{\theta^{\prime }}}\left(s\right)\right]\cdot E_{\mu_{s,a}}\left[\frac{\partial }{\partial \omega }\left(Q_{R_{\omega }}^{\pi_{\theta }}\left(s,a\right)-V_{R_{\omega }}^{\pi_{\theta }}\left(s\right)\right)\right].$$ (16)

By abbreviating the full and partial differentials, the gradient of the cumulative return term $J_{\mathrm{cum}}^{\text{upper}}$ with respect to the reward parameters ω can be expressed as

$$\nabla_{\omega }J_{\mathrm{cum}}^{\text{upper}}\left(\theta ,\omega \right)=E_{\mu_{s,a}}\left[\nabla_{\omega }\left[A_{R^{†}}^{\pi_{\theta }}\left(s,a\right)\cdot A_{R_{\omega }}^{\pi_{\theta }}\left(s,a\right)\right]\right].$$ (17)

The gradient operator acts only on $A_{R_{\omega }}^{\pi_{\theta }}\left(s,a\right)$.

The gradient of the penalty term with respect to the reward parameters can be succinctly written as

$$\nabla_{\omega }J_{\mathrm{pen}}^{\text{upper}}\left(\theta ,\omega \right)=E_{\mu_{s,a}}\left[\nabla_{\omega }\;d_{\cos}\left(A_{R_{\omega }}^{\pi_{\theta }}\left(s,a\right),A_{R^{†}}^{\pi_{\theta }}\left(s,a\right)\right)\right],$$ (18)

where $d_{\cos}\left(\cdot ,\cdot \right)$ denotes the cosine distance between the learned and prior advantage functions.

In summary, the overall gradient for upper-level optimization is obtained by combining the meta-gradient of the cumulative return and the analytic gradient of the penalty regularization term, which enables efficient joint optimization of the reward parameters in accordance with both the long-term task return and the prior-guided regularization.

### Training procedure

#### Reward function architecture

In practice, the reward function $R_{\omega }$ is implemented as a neural network model consisting of 3 main components: a state encoder, an action encoder, and a forward network. The state encoder transforms the full environmental state $s_{t}$ into a compact latent representation $z_{s}$, enabling the model to efficiently process high-dimensional sensory input. Simultaneously, the action encoder embeds the executed action $a_{t}$ into a lower-dimensional latent vector $z_{a}$, which facilitates flexible reward modeling across diverse action spaces. The outputs of the state and action encoders are concatenated and passed through a feed-forward module *F* that predicts the scalar reward, such that $R_{\omega }\left(s_{t},a_{t}\right)=F\left(\left[z_{s},z_{a}\right]\right)$. This architecture allows the reward model to fully exploit the available environmental information, capturing complex, context-dependent relationships that are critical for adaptive control.

#### Policy architecture

We use a standard PPO actor-critic framework. The actor and critic share the same network structure, consisting of 2 fully connected hidden layers with 64 units each. Policy optimization is performed using the clipped surrogate objective, with generalized advantage estimation for advantage computation. Training is carried out using the Adam optimizer with a buffer size of 2,048, a batch size of 64, and 10 optimization epochs per update. The learning rate is set to 3 × 10^−4^, with a discount factor $\gamma_{\mathrm{ppo}}=0.99$, generalized advantage estimation parameter $\lambda_{\mathrm{ppo}}=0.95$, clipping parameter ϵ=0.2, an entropy coefficient of 0.01, and a value loss coefficient of 0.5. All network architectures and hyperparameters are kept fixed across experiments to ensure reproducibility and fair comparison.

#### Alternating optimization procedure

The training process is organized as an alternating optimization routine, wherein the policy and reward models are updated in turn. At each iteration, the agent interacts with the environment to collect new trajectories $\xi =\left\{\;\left(o_{t},s_{t},a_{t},r_{t}\right)\;\right\}$, which are subsequently stored in a replay buffer for future sampling. The policy parameters are updated using the gradient of the lower-level objective $J^{\text{lower}}$, promoting behavior that is effective under the current reward function. Following each policy update, the reward parameters ω are optimized using the upper-level objective $J^{\text{upper}}$, ensuring that the learned reward continues to guide the policy toward task-relevant and interpretable behaviors. This alternating scheme is repeated until convergence, resulting in the coadaptation of both policy and reward networks. The pseudo-code of the proposed algorithm is provided in Algorithm 1.


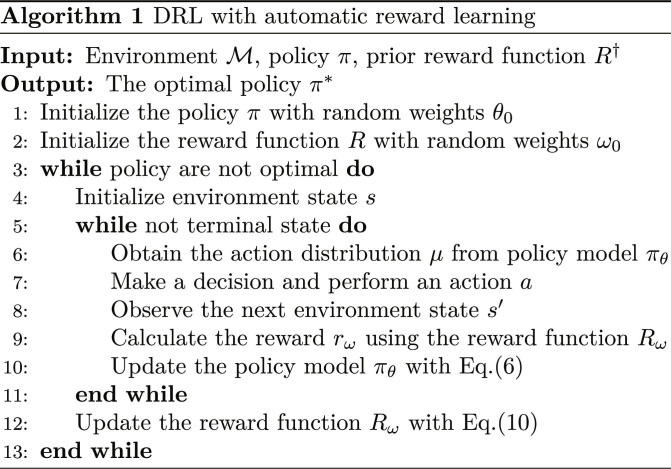


#### Monte Carlo estimation

Expectation terms appearing in the gradient-based updates for both the policy and the reward models are estimated via Monte Carlo sampling from the replay buffer. At each training step, batches of state, action, observation, and reward tuples are drawn to compute empirical estimates of returns, value functions, and advantage functions. Baseline value subtraction is applied to reduce the variance of gradient estimates, which is particularly important in partially observable and high-dimensional environments. This approach ensures that the learning signal remains robust and reliable throughout training and allows efficient utilization of previously collected experience.

#### Annealing of the regularization coefficient

To ensure the stability and reliability of the learned reward during the early stages of training, the alignment coefficient *λ* is initially set to a high value, which strongly emphasizes the influence of prior knowledge. This regularization prevents the learned reward function from deviating excessively and helps anchor the optimization process around semantically meaningful solutions defined by $R^{†}$. As training progresses and the policy acquires more experience, *λ* is gradually decreased according to a simulated annealing schedule. This reduction relaxes the constraint imposed by the prior, allowing the reward function to adapt more flexibly and capture the subtle, task-specific nuances required for optimal performance in the given environment. In this way, the learning process transitions smoothly from a prior-driven regime to one in which autonomous reward learning predominates, promoting both stability and adaptability throughout training.

## Data Availability

The data that support the findings of this study are generated by the learning agent through its interaction with the MuJoCo (https://mujoco.org/) and Isaac Lab simulation environments (https://developer.nvidia.com/isaac/lab/) and with the publicly available Unitree G1 robotic platform in the real-world experiments (https://support.unitree.com/home/en/G1_developer/). No static datasets are used in this work. Data access inquiries can be directed to rzlu@hust.edu.cn.
